# Treatment-free interval as an additional measure of efficacy in a large UK dataset of transplant ineligible myeloma patients

**DOI:** 10.1371/journal.pone.0229469

**Published:** 2020-02-21

**Authors:** Faouzi Djebbari, Faye A. Sharpley, Susan McLain-Smith, Grant Vallance, Toby A. Eyre, Jaimal Kothari, Sally Moore, Karthik Ramasamy

**Affiliations:** 1 Department of Clinical Haematology, Oxford University Hospitals NHS Foundation Trust, Oxford, United Kingdom; 2 PH Associates, Marlow, United Kingdom; European Institute of Oncology, ITALY

## Abstract

Treatment of transplant-ineligible (TNE) newly diagnosed multiple myeloma (NDMM) requires a balance between disease control and maintaining quality of life (QoL). Patients value treatment-free remission periods in this incurable condition, as they are associated with better QoL. We set out to study clinical outcomes of consecutive TNE NDMM patients in routine care treated in Thames Valley Cancer Network between 2009 and 2017. The primary outcome was the evaluation of the treatment-free interval (TFI) after 1st and subsequent lines of therapy in the total cohort and in individual subgroups, according to age (≤75 vs. >75 years), and co-morbidities using Charlson Co-morbidity Index (CCI): mild: 0–2 vs. moderate: 3–4 vs. severe: ≥5). Secondary outcomes include response rates, overall survival (OS) and progression-free survival (PFS) between subgroups: according to age and according to co-morbidities. In a total cohort of 292 patients, median TFI (IQR) was longest after first-line therapy 6.9 months (1.4–16.9), reducing after second line therapy to 1.8 months (.7–6.9), and after third line therapy to 0.6 months (0.2–1.5). Median TFI followed the same trend across the different subgroups, by age (≤75, >75 years) and by CCI (0–2, 3–4, ≥5). Overall response rate (ORR) to first line therapy for total cohort was 67%, with responses categorised as complete response (CR): 21%, very good partial response: 16%, partial response: 30%, stable disease: 18%, and progressive disease: 8%. ORR in individual subgroups by age were (≤75: 70% vs. >75: 63%), and by CCI (0–2: 65% vs. 3–4: 71% vs. ≥5: 77%). Median OS and PFS for the total cohort were (30.2 months, 95% CI: 23.8–36.9), and (9 months, 95% CI: 7.9–9.8), respectively. Patients aged >75 years showed a significant reduction in OS and PFS compared to those ≤75 years of age: OS (49.0 vs. 22.4 months, p<0.0001, HR: 2.08, 95% CI: 1.5–2.8), PFS (9.7 vs. 8.0 months, p<0.01, HR: 1.47, 95% CI: 1.1–1.9). Median OS was significantly reduced with worsening co-morbidities: (CCI 0–2: 52.4 months vs. CCI 3–4: 33.0 months vs. CCI ≥5: 24.0 months, p = 0.01, HR: 1.43, 95% CI: 1.1–1.9). Median PFS was significantly reduced in the severely co-morbid subgroup (CCI 0–2: 9.4 months vs. CCI 3–4: 9.6 months vs. CCI ≥5: 7.1 months, p = 0.025, HR: 1.3, 95% CI: 1.0–1.6). This study demonstrated that first line therapy in the TNE NDMM setting resulted in the longest TFI which was modest at a median of 6.9 months, and decreased significantly following subsequent lines of therapy and across the different subgroups by age and by co-morbidities. Therapy objective should be to maximise the benefit of first line treatment. We envisage that the recent shift towards a continuous therapeutic approach will benefit TNE patients in view of improved survival data demonstrated by a number phase 3 trials. When continuous therapy is not appropriate due to patient choice or toxicities, an efficacious (not limited to thalidomide and bortezomib) but tolerable first line FDT strategy, which can maximise TFI and maintain a good QoL, remains a reasonable alternative approach.

## Introduction

Multiple myeloma is primarily a disease of the elderly with up to 45% of new diagnoses in the UK made in patients aged 75 and over [1]. Age-specific incidence rates increase steadily in the 50–54 age group and more steeply in the 65–69 age group. The highest incidence rates in both males and females occur in the 85–89 age group [1].

Elderly myeloma patients are typically ineligible for autologous stem cell transplant (ASCT) due to advanced age and co-morbidities. Objectives of first-line treatment in this patient population are disease control whilst maintaining quality of life (QoL), which translates into improved survival [2].

As the myeloma treatment landscape continues to be shaped, continuous therapy has become the new standard of care. Progression-free survival (PFS) advantage of continuous lenalidomide with dexamethasone (Rd) was demonstrated in the MM015 trial and the FIRST trial [3, 4]. In addition, survival advantage of continuous daratumumab with bortezomib, melphalan and prednisolone (D-VMP) was recently demonstrated in the ALCYONE trial [5]. More recently, MAIA trial in the TNE NDMM setting demonstrated PFS advantage of continuous daratumumab with lenalidomide and dexamethasone (D-Rd) compared to continuous Rd alone [6].

However, the decision to employ a continuous first-line strategy in routine practice requires a careful account of a number of patient-related factors. At least 30% of patients are frail, due to disease-related symptoms and/or age-related decline in physical capacity in addition to co-morbidities, polypharmacy, nutritional status, and cognitive impairment [7]. Achieving optimal outcomes in newly diagnosed patients over 75 years of age remains a considerable challenge for the myeloma community.

Treatment free interval (TFI) in routine practice can occur in a planned or unplanned fashion. Physicians and patients often decide to plan for a fixed duration therapy (FDT) strategy based on response achieved from therapy. In addition, upfront therapy can be discontinued in an unplanned fixed duration fashion as a result of significant toxicities leading to early treatment discontinuation. In both scenarios, TFI becomes an issue of immense importance to patients and clinicians, and can allow patients to recover from toxicities and restore a good QoL.

It is, therefore, important to tailor the choice and duration of therapy to patients’ priorities and individual needs. Functional status and QoL are primary objectives in this population [2]. Health-related QoL in myeloma is primarily influenced by therapy, which on one hand can improve disease-related symptoms, but on the other hand can result in significant toxicities and a subsequent deterioration in QoL. An additional analysis from the FIRST trial demonstrated that continuous Rd achieves superior health-related QoL during treatment, compared with melphalan, prednisone, thalidomide (MPT) [8].

TFI and good QoL have been described as additional measures of efficacy which can be employed to make individualised treatment decisions. A UK cross-sectional survey of 370 myeloma patients demonstrated that being in a first TFI and experiencing a longer TFI were significantly associated with a better HRQL as assessed by various domains of the QLQ-C30, MY20 and EQ-5D [9].

UK’s National Institute for Health and Care Excellence (NICE) recently approved lenalidomide with dexamethasone as first-line therapy in TNE NDMM patients where thalidomide is not tolerated or inappropriate [10]. Prior to this, FDT was the standard of care in this patient population of either a bortezomib-based or a thalidomide-based regimen, for the first two lines of therapy, and remains an option if lenalidomide is inappropriate.

In view of the recent shift towards continuous therapy, we looked to evaluate the TFI as an additional metric of efficacy in routine practice, after 1st and subsequent lines of therapy, in a large cohort of TNE NDMM patients. To our knowledge, there are no published data quantifying TFI exclusively in the TNE NDMM setting.

We have also assessed the influence of age (<75 vs. ≥75 years) and co-morbidities (as per Charlson Co-mordibity Index: CCI) on clinical outcomes. The aim was to understand current practice and identify strategies which can increase first TFI and improve future outcomes.

## Materials and methods

### Study design, inclusion criteria and data collection

TNE NDMM patients are defined as those with a new diagnosis of symptomatic multiple myeloma which requires initiation of systematic first line therapy, but who are not eligible for, and did not receive a autologous stem cell transplantation (ASCT) due to age and/or co-morbidities. All patients with TNE NDMM within the UK Thames Valley Cancer network with measurable disease, as defined by International Myeloma Working Group (IMWG) guidelines, treated with at least one cycle of systemic chemotherapy between 2009 and 2017 were eligible for inclusion. Patients were excluded if they had been treated as part of a clinical trial. The study was approved locally by the Clinical Governance Committee.

Data was retrospectively collected from patients’ medical and chemotherapy records including baseline demographic and clinical characteristics (including age, sex, date of diagnosis, International Staging System [ISS], myeloma isotype, renal function, and Charlson Co-morbidity Index (CCI)), anti-myeloma treatment (including treatment type, duration and the mean dose of first-line treatment). All patients consented for retrospective analysis of their records at the point of treatment, and all patient records were anonymised at the point of analysis.

### Study endpoints

The primary endpoint was the evaluation of treatment-free interval (TFI) following 1st and subsequent lines of therapy in the total cohort, and the following subgroups: according to age (≤75 vs. >75 years) and according to co-morbidities: (CCI: 0–2) vs. (CCI: 3–4) vs (CCI: ≥5). TFI was evaluated as the time between the last dose of one line of treatment and the first dose of a subsequent treatment. Response rates for the total cohort were also evaluated after first line therapy.

Secondary outcomes include response rates, OS and PFS between subgroups: according to age (≤75 vs. >75 years) and according to CCI.

OS was defined as time from initiation of first anti-myeloma treatment to death from any cause. PFS was evaluated as the time between initiation of first anti-myeloma treatment and progressive disease (based on IMWG uniform response criteria) or death. Patients who did not receive another anti-myeloma therapy were censored at the last available date the patient was known to be alive.

### Statistical analyses

Descriptive statistics for quantitative variables are presented as mean (standard deviation [SD]) or median (interquartile range [IQR] and/or range). Descriptive statistics for categorical variables are presented as number (%). Time-dependent variables were evaluated and presented using the Kaplan-Meier method and reported as median (95% confidence intervals [95% CI]). Time-dependent variables were compared between the different age groups (≤75 years vs. >75) CCI co-morbidity subgroups (mild: 0–2 vs. moderate: 3–4 vs. severe: ≥5) using unstratified log-rank tests and Cox regression analyses, with proportionality of hazards evaluated using Schoenfeld residuals, and hazard ratios (HR) presented with 95% CI.

## Results

### Patient demographic and clinical characteristics

A total of 292 patients were eligible for inclusion. The baseline demographic and clinical characteristics of the total cohort as well subgroups (≤75 vs. >75 years, CCI: 0–2 vs. 3–4 vs. ≥5) are presented in Table 1. The median ages (IQR) were: 75.1 (69 to 81) for total cohort, 68.6 (63 to 72) for ≤75 year group, 80.7 (78 to 84) for >75 years group, 62.1 (57 to 67) for CCI (0–2) group, 75.9 (72 to 81) for CCI (3–4) group and 77.9 (74 to 83) for CCI (≥5) group. Distributions of gender and combination therapy regimen were comparable across subgroups aged ≤75 vs >75 years and across the different co-morbidity subgroups.

**Table 1:** Patient and clinical characteristics.

		Total patient cohort	Subgroups by age		Subgroups by Charlson Co-morbidity Index (CCI)		
**Variable**		**(*n* = 292)**	**Age ≤75 (*n* = 144)**	**Age >75 (*n* = 148)**	**CCI 0–2 (n = 41)**	**CCI 3–4 (n = 92)**	**CCI ≥5 (n = 52)**
**Age** (years)^a^		75.1 (69 to 81)	68.6 (63 to 72)	80.7 (78 to 84)	62.1 (57 to 67)	75.9 (72 to 81)	77.9 (74 to 83)
**Male**^b^		165 (57%)	80 (56%)	85 (57%)	23 (56%)	44 (48%)	36 (69%)
**ISS stage**^b^							
I		54 (18%)	38 (26%)	16 (11%)	15 (37%)	19 (21%)	4 (8%)
II		46 (16%)	25 (17%)	21 (14%)	10 (24%)	17 (18%)	7 (13%)
III		140 (48%)	60 (42%)	80 (54%)	9 (22%)	46 (50%)	37 (71%)
Unknown		52 (18%)	21 (15%)	31 (21%)	7 (17%)	10 (11%)	4 (8%)
**Serum creatinine ≥ 140 μmol/L**^b^		(*n* = 292) 82 (28%)	(*n* = 144) 39 (27%)	(*n* = 148) 43 (29%)	(*n* = 41) 3 (7%)	(*n* = 92) 20 (22%)	(*n* = 52) 25 (48%)
**Charlson co-morbidity index**	0–2: mild	41 (14%)	39 (27%)	2 (1%)			
	3–4:moderate	92 (32%)	42 (29%)	50 (34%)			
	≥5: severe	52 (18%)	14 (10%)	38 (26%)			
	NK	107 (37%)	49 (34%)	58 (39%)			
**First line treatment**	THAL-based	178 (61%)	82 (57%)	96 (65%)	5 (12%)	68 (74%)	28 (54%)
	PI-based	64 (22%)	42 (29%)	22 (15%)	0 (0%)	13 (14%)	16 (31%)
	LEN-based	20 (7%)	16 (11%)	4 (3%)	9 (22%)	3 (3%)	0 (0%)
	Alkylator-based	30 (10%)	4 (3%)	26 (18%)	27 (66%)	8 (9%)	8 (9%)
**Combination regimen**^b^		(*n* = 292)	(*n* = 144)	(*n* = 148)	(*n* = 41)	(*n* = 92)	(*n* = 52)
	Doublet	88 (30%)	45 (31%)	43 (29%)	13 (32%)	18 (20%)	21 (40%)
	Triplet	204 (70%)	99 (69%)	105 (71%)	28 (68%)	74 (80%)	31 (60%)
**First-line therapy cycles**^a^		(*n* = 286) 6 (3 to 6)	(*n* = 140) 6 (3 to 6)	(*n* = 146) 6 (3 to 6)	(*n* = 41) 6 (4 to 7)	(*n* = 92) 6 (4 to 6)	(*n* = 52) 5 (3 to 6)

Data presented as: *median (interquartile range [IQR]) or **n (%), ***Cytogenetics data [high risk patient numbers: 6 with p53 loss, 3 with t(4;14) and 6 with t(14;20); non-high risk: 14 with 1q gain, 10 with t(11;14) and 1 with 1p32 loss; 10 with nil cytogenetic abnormalities; 258 with no known cytogenetic data. ISS: International Staging System; NK: not known data; THAL: thalidomide; PI: proteasome inhibitor (bortezomib); LEN: lenalidomide.

At presentation, 48% of patients in the total cohort had an International Staging System (ISS) of III. A meaningful analysis of outcomes based on cytogenetics was not possible due to lack of data. The proportion of patients with renal impairment (serum creatinine ≥140 μmol/L) were comparable between the two age subgroups (≤75 years: 27%, >75 years: 29%), but renal impairment was more prevalent in the severely co-morbid subgroup (CCI 0–2: 7% vs. CCI 3–4: 22% vs. CCI≥5: 48%).

The older cohort (>75 years) presented with a significantly higher proportion of moderately to severely co-morbid patients compared to the younger cohort (60% vs. 39%).

Within the total cohort, thalidomide was the most commonly used first line therapy (61%), followed by PI-based (bortezomib) (22%), alkylator-based (10%), and lenalidomide-based (7%). Bortezomib was only approved for use as first line in the UK in 2014, which explains the smaller patient numbers compared to THAL group. The mean daily thalidomide dose was 51.4mg (range 0-133mg). The mean cumulative bortezomib dose per cycle was 4 mg/m^2^ (range 0.7–5.2 mg/m^2^), Table 2.

**Table 2:** Dose intensity of thalidomide and bortezomib at first line therapy.

Dosing of first line therapy	THAL group (N = 144)*	Bort (PI) group (N = 49)**
	Daily thalidomide dose (mg)	Cumulative botezomib dose per cycle (mg/m2)
**Mean**	51.37	4.03
**SD**	23.3	1.1
**Median**	50	3.9
**Lower quartile 25%**	50.0	3.7
**Upper quartile 75%**	52	5.0
**Min**	0	1
**Max**	133.3	5.2
**IQR**	50.0 to 51.8	3.7 to 5.0
**Range**	0.0 to 133.3	0.7 to 5.2

*data unknown in 34 patients.

**data unknown in 15 patients

The mean number of treatment cycles was comparable (6 cycles) for the total cohort as well as individual subgroups according to age or according to co-morbidities. Except for only 20 patients in the total cohort who received lenalidomide until disease progression or unacceptable toxicity, the rest of patients in this large study were treated with FDT in a pre-planned fashion.

Table 3 shows the different treatment regimen used as first line therapy. Physicians’ choice of triplet over doublet regimen was comparable between age groups (≤75: 69%, >75years: 71%). The use of triplets over doublets was also evident in the different co-morbidity subgroups (CCI 0–2: 68% vs. CCI 3–4: 80% vs. CCI ≥5: 60%). Median duration of follow up for the total patient cohort was 22.5 months (IQR 11.2 to 41.1). Median duration of first-line FDT for the whole cohort was 4.6 months (IQR: 2.5–5.7).

**Table 3:** Chemotherapy regimen used as first line therapy.

	1^st^ line Treatment regimen	Patient numbers (%) and duration of treatment		
		n	%	Median duration (months)
**Thalidomide-based**	CTDa	97	33%	5.1
	CTD	49	17%	4.3
	MPT	26	9%	4.6
	TD	5	2%	4.6
	BTD	1	0%	1.0
**Bortezomib-based**	VCD	14	5%	4.1
	VD	42	14%	3.6
	VMP	5	2%	3.9
	VTD	2	1%	0.7
**Chemo-based**	CD	1	0%	12.7
	CP	9	3%	3.9
	MP	21	7%	3.9
**Lenalidomide-based**	RCD	3	1%	7.0
	RCDa	6	2%	5.3
	RD	11	4%	11.8
	**Total**	**292**	**100%**	

CTDa (attenuated cyclophosphamide with thalidomide and dexamethasone), CTD (cyclophosphamide with thalidomide and dexamethasone), MPT (melphalan with prednisolone and thalidomide), TD (thalidomide with dexamethasone), BTD (bendamustine with thalidomide and dexamethasone), VCD (bortezomib with cyclophosphamide and dexamethasone), VD (bortezomib and dexamethasone), VMP (bortezomib with melphalan and prednisolone), VTD (bortezomib with thalidomide and dexamethasone), CD (cyclophosphamide with dexamethasone), CP (cyclophosphamide with prednisolone), MP (melphalan with prednisolone), RCD (lenalidomide with cyclophosphamide and dexamethasone), RCDa (attenuated RCD), RD (lenalidomide with dexamethasone).

### Response rates to 1^st^ line therapy

Overall response rates for total cohort as well as individual subgroups are presented in Table 4. Overall response rate (ORR) for total cohort was 67%, with responses categorised as CR: 21%, VGPR: 16%, PR: 30%, SD: 18%, PD: 8% and unknown 7%. ORR in individual subgroups according to age were(≤75: 70% vs. >75: 63%), and according to co-morbidities were (CCI 0–2: 65% vs. CCI 3–4: 71% vs. CCI≥5: 77%).

**Table 4:** Response rates to first line therapy.

Response to first-line therapy	Total cohort (*n* = 292)	Age subgroups		Charlson Co-morbidity Index subgroups		
		Age ≤75 (*n* = 144)	Age >75 (*n* = 148)	CCI 0–2 (n = 41)	CCI 3–4 (n = 92)	CCI ≥5 (n = 52)
**ORR**	67%	70%	63%	65%	71%	77%
**Best response**						
CR	60 (21%)	38 (26%)	22 (15%)	14 (34%)	18 (20%)	11 (21%)
VGPR	46 (16%)	23 (16%)	23 (16%)	3 (7%)	13 (14%)	14 (27%)
PR	87 (30%)	40 (28%)	47 (32%)	10 (24%)	34 (37%)	15 (29%)
SD	54 (18%)	22 (15%)	32 (22%)	9 (22%)	17 (18%)	7 (13%)
PD	24 (8%)	13 (9%)	11 (7%)	5 (12%)	7 (8%)	4 (8%)
NK	21 (7%)	8 (6%)	13 (9%)	0 (0%)	3 (3%)	1 (2%)

Data presented as % or n (%). CCI: Charlson Co-morbidity Index. ORR: overall response rate; CR: complete Response; VGPR: very good partial response; PR: partial response; SD: stable disease; PD: progressive disease; NK: not known.

### Treatment-free interval (TFI)

**Fig 1:**
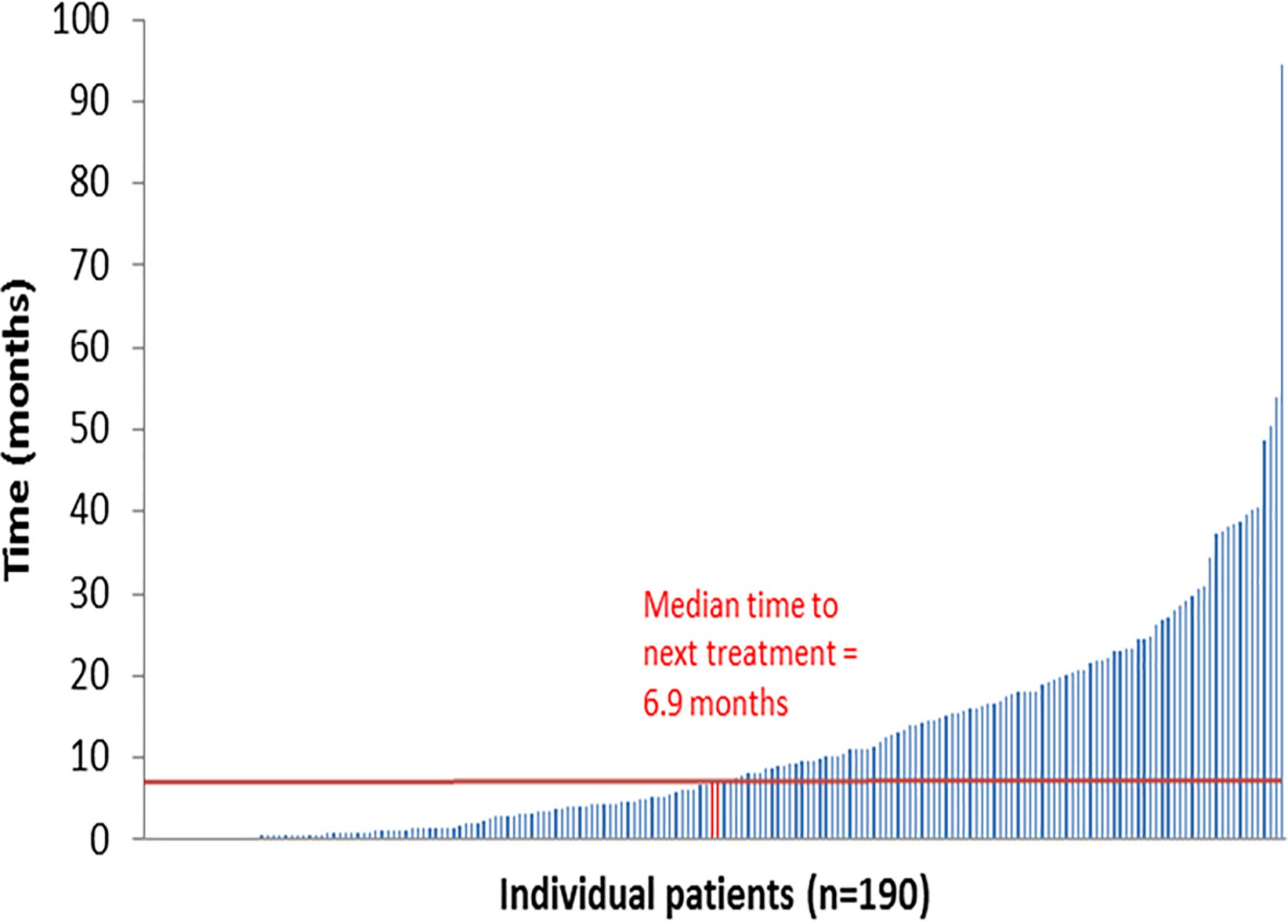
Waterfall plot of treatment-free interval following first-line therapy.

In the total cohort, median (IQR) TFI was longest after first-line therapy 6.9 months (IQR: 1.4–16.9, n = 190) Fig 1. Treatment-free interval reduced after second line therapy to 1.8 months (IQR: 0.7–6.9, n = 101), and after third line therapy to 0.6 (IQR: 0.2–1.5, n = 38), Table 5.

**Table 5:** Treatment-free interval in the total cohort following first-line and subsequent lines of therapy.

Treatment-free interval (months)	End Trt 1 to Start Trt 2	End Trt 2 to Start Trt 3	End Trt 3 to Start Trt 4	End Trt 4 to Start Trt 5
N	190	101	38	20
Mean	11.3	5.8	1.9	2.0
SD	13.0	8.4	3.4	3.0
Median	6.9	1.8	0.6	0.8
IQR	1.4 to 16.9	0.7 to 6.9	0.2 to 1.5	0.4 to 2.1
Range	0.0 to 94.6	0.0 to 39.8	0.0 to 15.4	0.0 to 10.5

Trt (treatment), SD: standard deviation, IQR (interquartile range).

TFI in individual subgroups according to age were also longest after first line therapy, and decreased with increasing lines of therapy: ≤75 years (median decreased from 7.5 months to 1.8 then to 0.5 months), >75 years (median decreased from 6.1 months to 2.1 then to 1.2 months). Table 6.

**Table 6:** Treatment-free interval in months according to age, following first-line and subsequent lines of therapy.

	≤75 years				>75 years			
	End Trt 1 to Start Trt 2	End Trt 2 to Start Trt 3	End Trt 3 to Start Trt 4	End Trt 4 to Start Trt 5	End Trt 1 to Start Trt 2	End Trt 2 to Start Trt 3	End Trt 3 to Start Trt 4	End Trt 4 to Start Trt 5
N	109	65	30	17	81	36	8	3
Mean	12.9	6.0	1.6	2.1	9.0	5.3	2.8	1.1
SD	15.3	8.8	3.3	3.2	8.6	7.6	3.9	1.9
Median	7.5	1.8	0.5	0.9	6.7	2.1	1.2	0.0
IQR	1.4 to 18.9	0.8 to 7.5	0.2 to 1.2	0.4 to 1.8	1.4 to 14.6	0.6 to 5.5	0.6 to 2.9	0.0 to 1.6
Range	0.0 to 94.6	0 to 39.8	0 to 15.4	0 to 10.5	0 to 38.8	0 to 32.7	0.3 to 12	0.0 to 3.3

Trt (treatment), SD: standard deviation, IQR (interquartile range).

TFI in individual subgroups according to co-morbidities were also longest following first line therapy and decreased with increasing lines of therapy: In the mildly co-morbid group (CCI = 0–2), median TFI decreased from 4.8 months to 1.9 then to 0.4 months. In the moderately co-morbid group (CCI = 3–4), median TFI decreased from 6.9 months to 2 then to 0.5 months. In the severely co-morbid group (CCI≥5), median TFI decreased from 8.9 months to 1 month then increased to 3.2 months following 3^rd^ line therapy, Table 7.

**Table 7:** Treatment-free interval in months according to co-morbidities following first-line and subsequent lines of therapy.

	CCI 0–2			CCI 3–4			CCI ≥5		
	End Trt 1 to Start Trt 2	End Trt 2 to Start Trt 3	End Trt 3 to Start Trt 4	End Trt 1 to Start Trt 2	End Trt 2 to Start Trt 3	End Trt 3 to Start Trt 4	End Trt 1 to Start Trt 2	End Trt 2 to Start Trt 3	End Trt 3 to Start Trt 4
**N**	31	21	10	65	38	15	31	14	6
**Mean**	12.5	7.2	0.9	11.2	5.7	0.6	11.4	3.8	5.7
**SD**	14	10.2	1.5	12.4	7.7	0.5	9.8	6.1	6
**Median**	4.8	1.9	0.4	6.9	2	0.5	8.9	1	3.2
**IQR**	1.2–22.3	0.9–7.9	0.1–0.9	1.9–14.8	0.8–6.8	0.2–0.7	3.2–19.7	0.3–5.2	1.5–9.0
**Range**	0.0–53.9	0.1–39.8	0.0–5.1	0.0–50.5	0.0–32.7	0.0–1.5	0.0–30.7	0.0–21.9	0.6–15.4

CCI (Charlson Co-morbidity Index) Trt (treatment), SD: standard deviation, IQR (interquartile range).

### Survival

**Fig 2:**
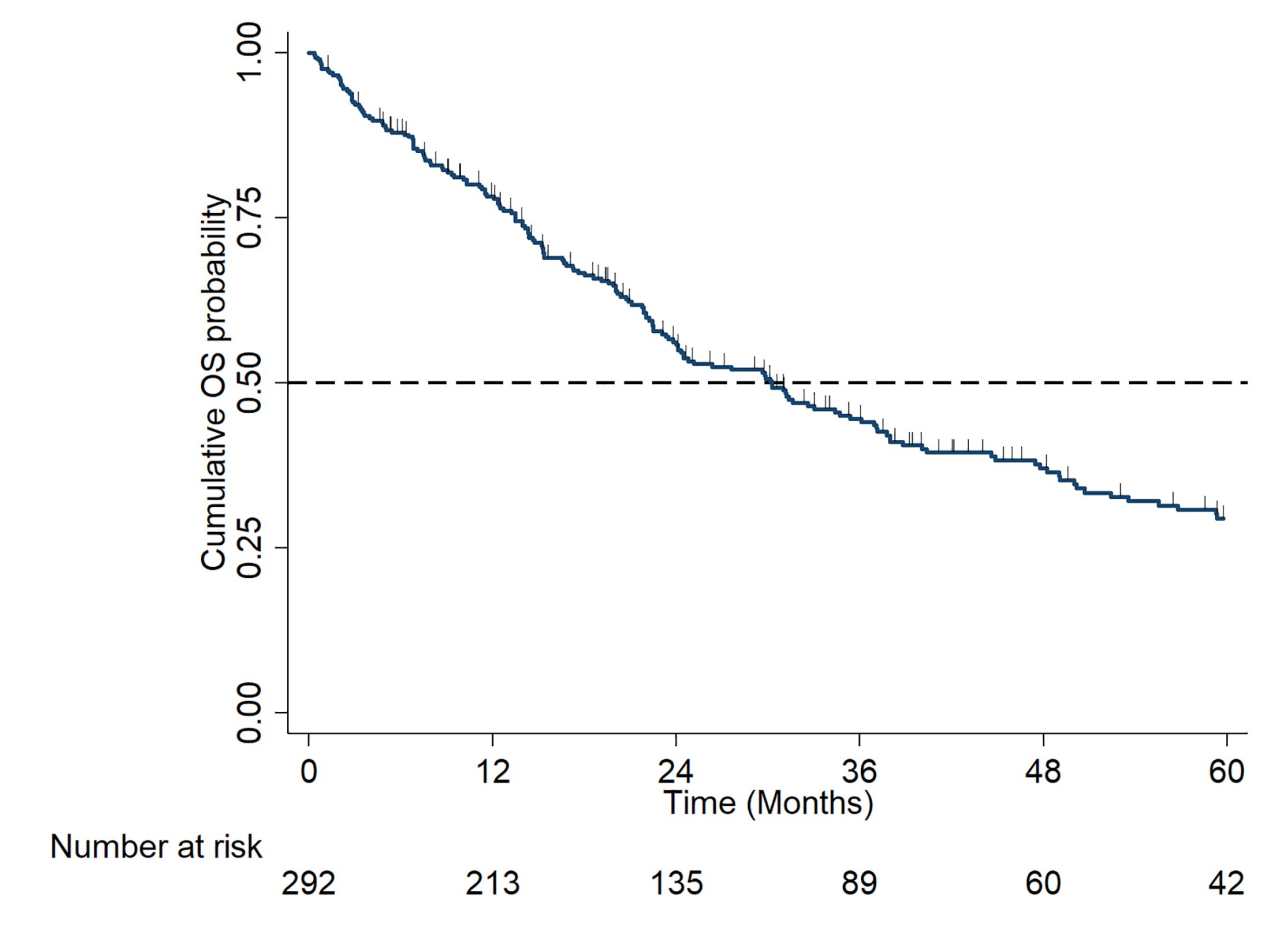
Kaplan Meier Curve of overall survival (OS) in the total cohort.

**Fig 3:**
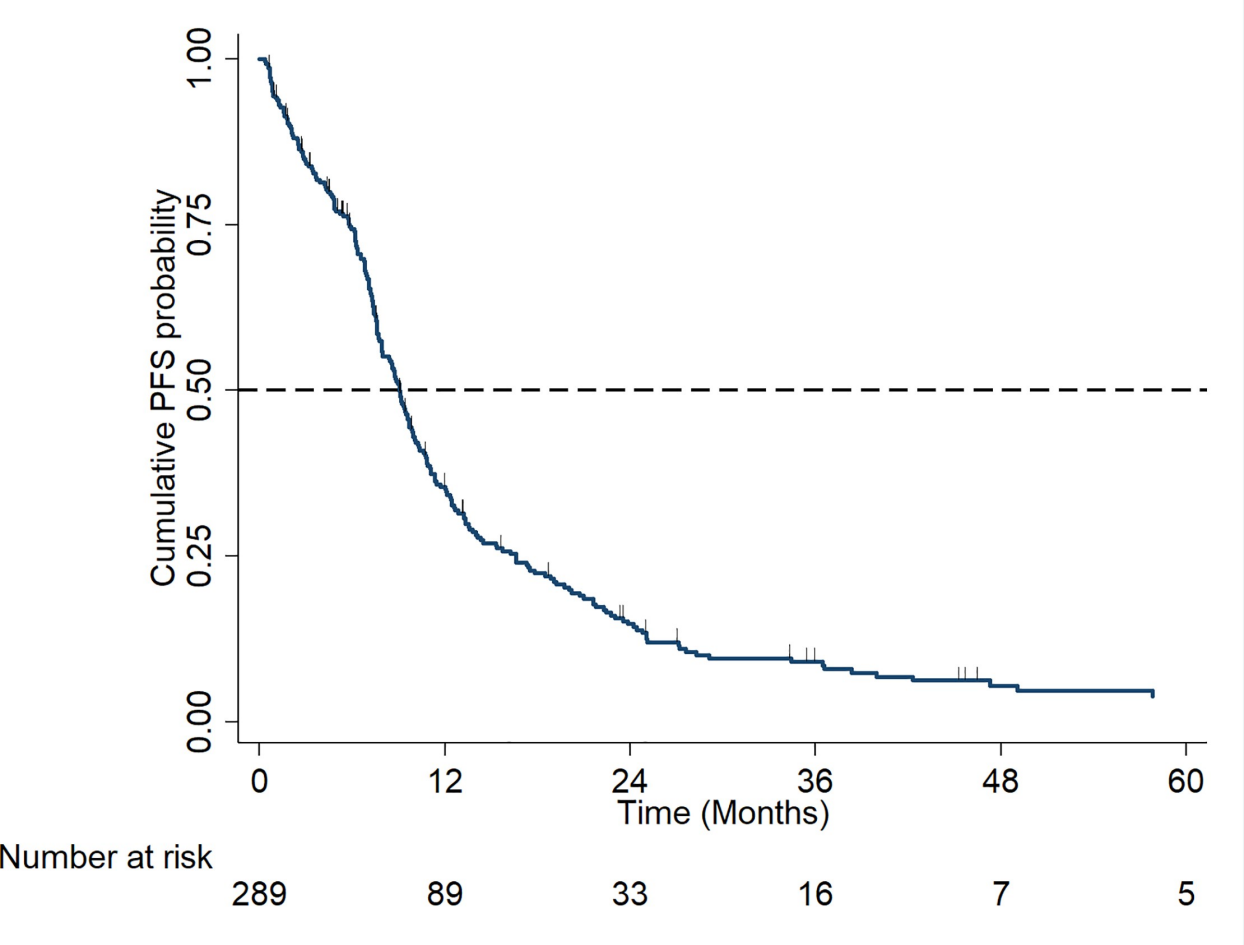
Kaplan Meier Curve of progression-free survival (PFS) in the total cohort.

Median OS for the total cohort was 30.2 months (95% CI: 23.8–36.9), Fig 2. Median PFS for the total cohort was 9.0 months (95% CI: 7.9–9.8), Fig 3. As per Table 3, poor PFS outcomes are partly due to choice of available therapies in this cohort: CTD (cyclophosphamide with thalidomide and dexamethasone) or attenuated CTD in 50% of patients, and VD (bortezomib and dexamethasone doublet) in 14% of patients. In addition, this cohort is characterised by advanced age and co-morbidities, which can influence dose intensity and duration of therapy.

**Fig 4:**
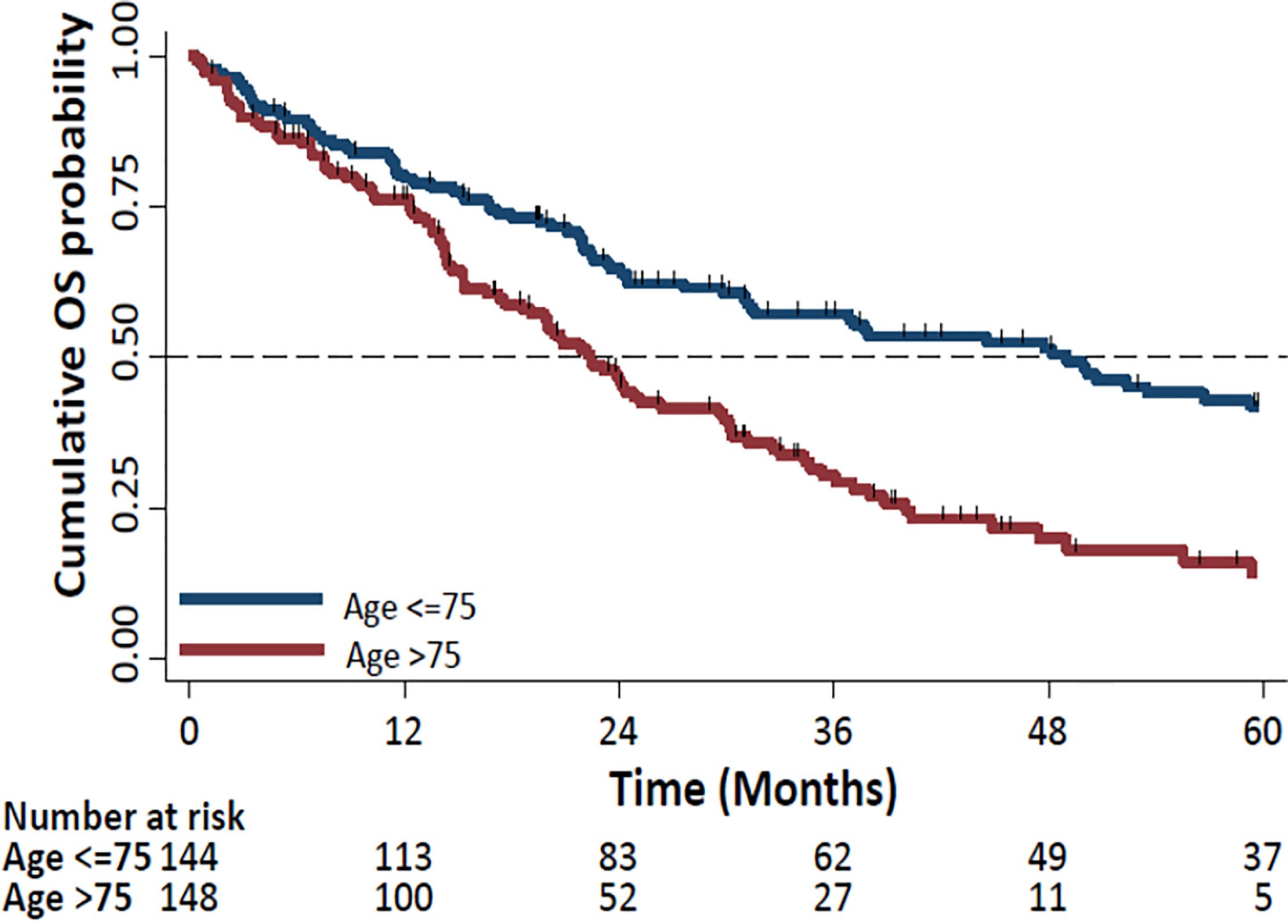
Kaplan Meier Curves of overall survival (OS) by age (>75 vs. ≤75 years).

OS was significantly longer in the ≤75 years group (49.0 months (95% CI: 31.3–61.8) compared to the >75 years group (22.4 months (95% CI: 18.6–29.6)), P<0.0001, HR: 2.08 (95% CI: 1.5–2.8), Fig 4. Survival rates in patients aged ≤75 vs. >75 years at 1 year were 80.4% vs. 75.9%, respectively, and at 2 years were 64.8% vs. 46.2%, respectively.

**Fig 5:**
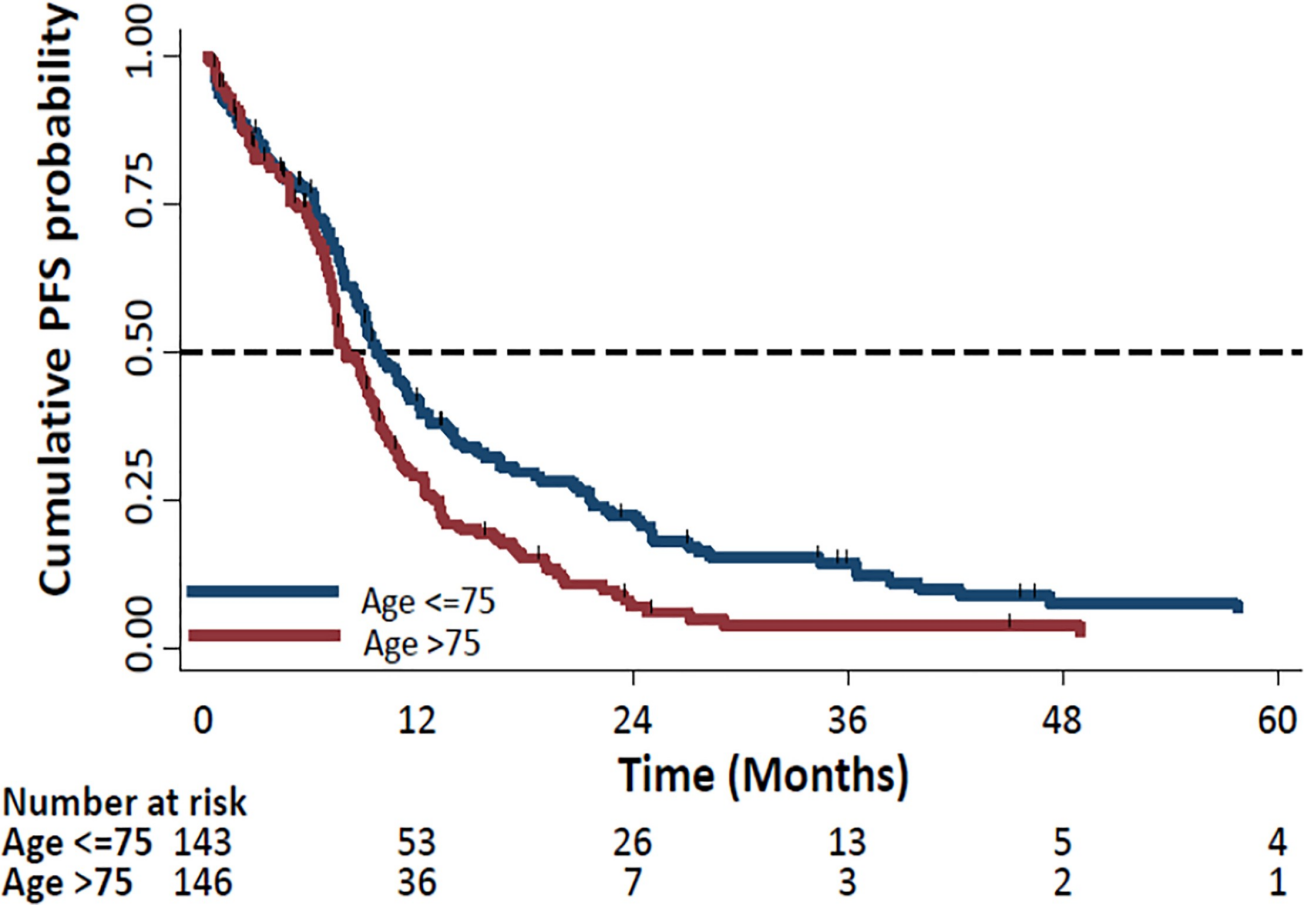
Kaplan Meier Curves of progression-free survival (PFS) by age (>75 vs. ≤75 years).

PFS was significantly longer in the ≤75 years group (9.7 months (95% CI: 8.6–12.0)) compared to the >75 years group (8.0 months (95% CI: 7.4–9.4)), P<0.01, Hazard ratio: 1.47 (95% CI: 1.1–1.8), Fig 5.

**Fig 6:**
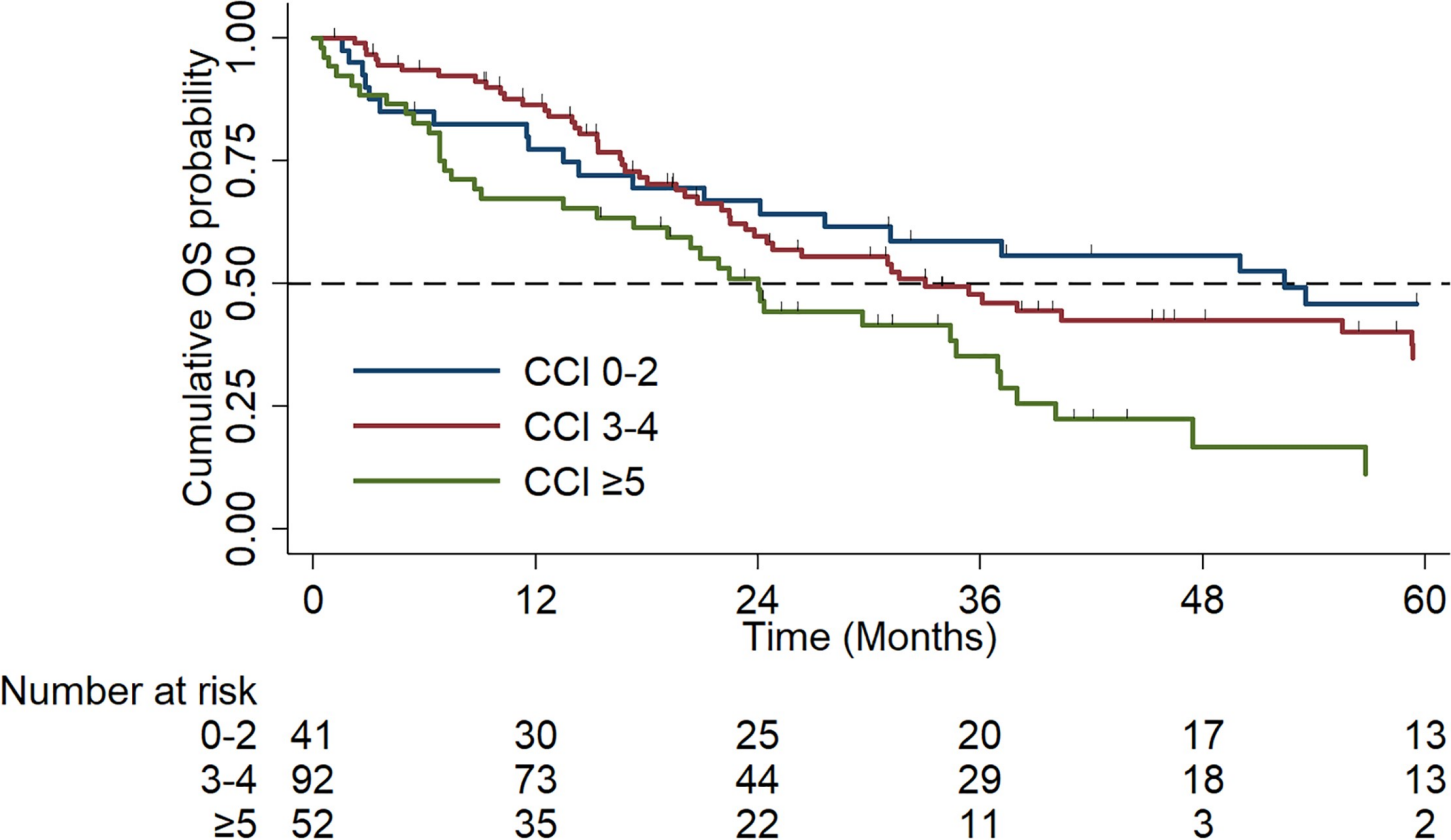
Kaplan Meier Curves of overall survival (OS) by Co-morbidity Index (mild (0–2) vs. moderate (3–4) vs. severe (≥5)).

**Fig 7:**
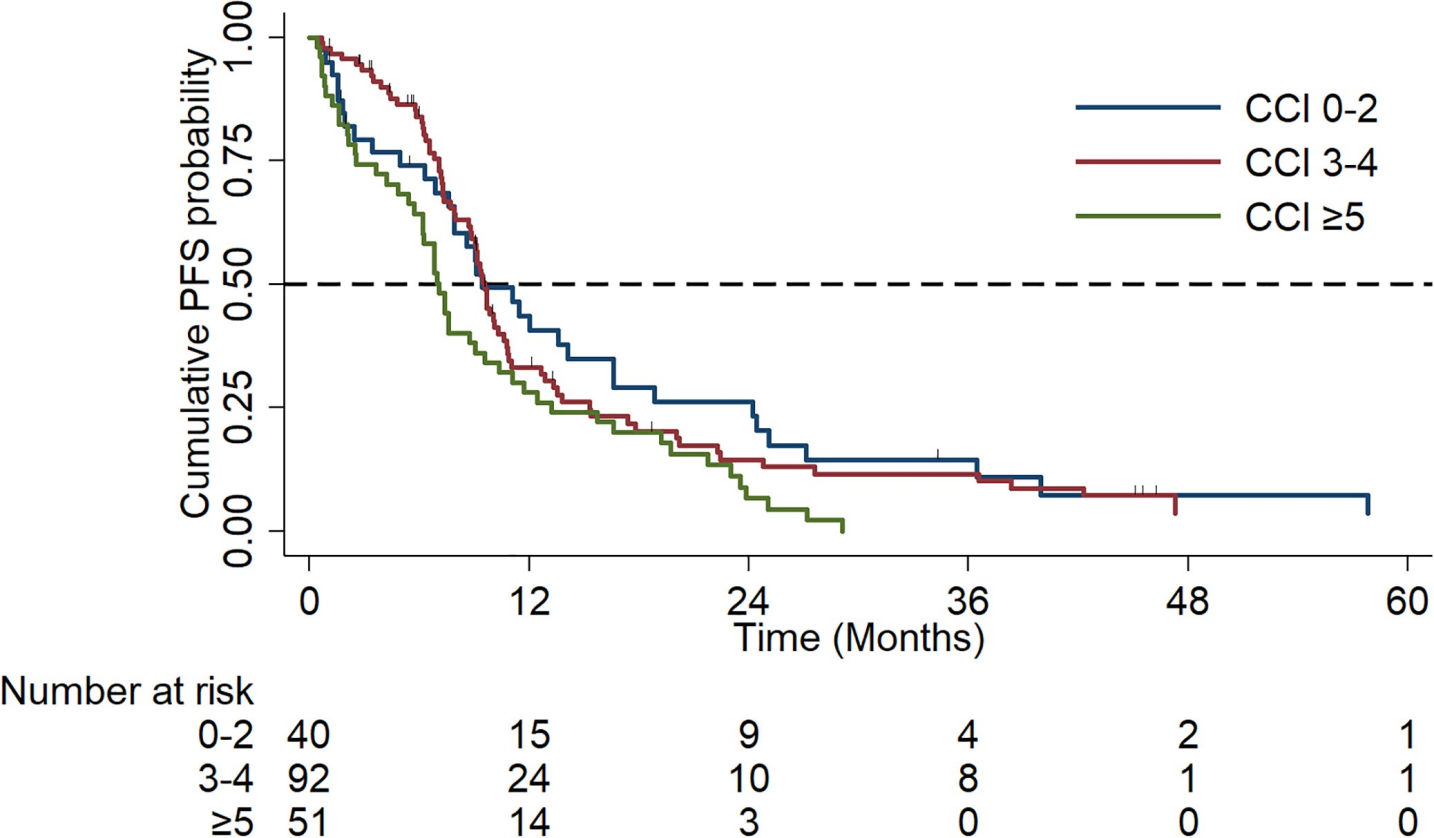
Kaplan Meier Curves of progression-free survival (PFS) by Co-morbidity Index (mild (0–2) vs. moderate (3–4) vs. severe (≥5)).

Median OS was significantly reduced with worsening co-morbidity index: (CCI 0–2: 52.4 months vs. CCI 3–4: 33.0 months vs. CCI ≥5: 24.0 months, p = 0.01, HR: 1.43, 95% CI: 1.1–1.9), Fig 6. Median PFS was significantly reduced in the severely co-morbid subgroup (CCI 0–2: 9.4 months vs. CCI 3–4: 9.6 months vs. CCI ≥5: 7.1 months, p = 0.025, HR: 1.3, 95% CI: 1.0–1.6), Fig 7.

## Discussion

This large retrospective study of 292 patients suggests that TNE NDMM patients are able to achieve a good ORR (67%) in routine care, and this is despite a short median duration of treatment (4.6 months), older age and higher rates of co-morbidity. However, the median TFI in the total cohort was only 6.9 months and declined with subsequent treatment phases (reducing to 1.8 and 0.6 months after second and third line therapy, respectively). These findings are consistent with a large international real-world study of 4997 myeloma patients, which suggested that median TFI and time to progression decrease with increasing lines of therapy (1^st^ line: 10 months, 2^nd^ line: 5 months, 3^rd^ line: 3 months, 4^th^ line: 1 month) [11]. Our cohort showed a shorter TFI compared to the international study because we had an exclusive TNE NDMM population. In addition, the median duration of frontline therapy in the international study was longer than ours (6 months vs 4.6 months). Despite this, our study highlighted the significance of first-line therapy, which affords patients the longest TFI.

When we examined the different subgroups, by age (≤75 years vs. > 75 years), and according to co-morbidities (CCI: 0–2 vs. 3–4 vs. ≥5), our study also demonstrated that TFI was longest after first line therapy and decreased significantly following second and subsequent lines of therapy.

The cross-sectional study of 370 patients was the first UK-based myeloma study to report the impact of the first TFI on HRQoL. Overall, the results demonstrated that being in the first TFI was associated with better HRQL compared to later treatment lines, and that longer TFI were largely associated with better HRQL [9]. In another myeloma patient survey based in Germany, treatment preferences of 282 patients throughout their myeloma treatment journey were evaluated [12]. Treatment-free intervals, as well as an improved emotional quality of life were valued as therapy goals.

TFI has also been previously identified as a metric of patient experience and a valuable health outcome in the relapsed myeloma setting, as demonstrated in an additional analysis of Panorama-1 trial [13].

Our study findings also suggest that a FDT approach for patients >75 years confers an inferior PFS and OS compared to patients ≤75 years. Our data is consistent with a number of subgroup analyses from large phase 3 trials in the TNE NDMM setting which demonstrated evidence of poorer survival outcomes for patients aged >75 years compared to their younger counterparts (≤75 years). In the FIRST trial, patients aged >75 years benefited from continuous Rd versus MPT; however, PFS and OS periods were shorter for the older versus young group (PFS, 20.3 vs. 28.1 months; OS, 52.3 vs. 60.9 months) [3]. In VISTA trial, which compared VMP to MP; OS benefit was observed from VMP but this was less marked in the >75 years group compared to ≤75 years (43.3 months vs. not reached). PFS comparison between age groups was not reported [14]. In the recent ALCYONE trial, D-VMP showed a PFS advantage over VMP [5]. Therefore the poorer outcomes in >75 year old cohort is more likely to be due to a number of other factors rather than the duration of frontline therapy alone. Frailty, concurrent co-morbidities and increased therapy discontinuation rates are commonly recognised factors leading to worse survival outcomes in the older patients [7].

Our study demonstrated that OS and PFS were significantly shorter with worsening co-morbidity (as assessed by CCI categories: mild, moderate and severe). The Greek Myeloma Group showed that performance status was an independent prognostic factor influencing survival in an octogenarian myeloma cohort [15]. A retrospective study from Japan also showed that comorbidity burden and performance status were predictive of outcomes in a cohort of patients aged 80 and over [16].

In our large dataset, 50% of patients were moderately to severely co-morbid which partly explains poorer outcomes of therapy particularly in >75 years, and the short median duration of first line treatment (4.6 months).

Our data demonstrated that the use of FDT with a thalidomide or a bortezomib-based regimen was associated with modest PFS and OS outcomes particularly in patients >75 years. This FDT strategy also resulted in a short median TFI. We envisage that the shift towards a continuous therapy approach will benefit TNE NDMM patients as demonstrated by survival advantage in large phase 3 trials, and should be standard of care in order to improve outcomes.

FIRST trial demonstrated that median PFS in the continuous lenalidomide and dexamethasone arm (Rd) was significantly superior to 18 cycles of Rd and to FDT with melphalan in combination with prednisolone and thalidomide (MPT) (25.5 vs. 20.7 vs. 21.2 months, P<0.001). The 4-year OS rates were (continuous Rd: 59% vs. Rd18: 56% vs. MPT: 51%) [3]. MM-015 trial showed that melphalan in combination with prednisolone and lenalidomide followed by lenalidomide maintenance (MPR-R) significantly improved PFS compared to MPR or MP (31 vs. 14 vs. 13 months, P<0.001), with the greatest benefit observed in patients aged 65 to 75 years. The 3-year OS rates were (MPR-R: 70% vs. MPR: 62% vs. MP: 66%) [4]. MAIA trial showed a 30 month PFS rate of 70.6% for continuous daratumumab in combination with Rd (D-Rd) compared to 55.6% with Rd alone (P<0.001) [6]. Complete response rates were 47.6% and 24.9% for D-Rd and Rd groups, respectively (P<0.001) [6].

In patients with advanced age or with co-morbidities, tolerable dosing and close monitoring are required to limit toxicities and maintain continuous therapy.

If the continuous therapy option is not available, or not appropriate (due to toxicities or patient choice), an effective FDT combination containing novel agents (not limited to thalidomide or bortezomib) and with good tolerability remains a reasonable alternative approach. FIRST trial showed no overall survival benefit between continuous Rd and 18 cycles of Rd (Benboubker, 2014). In certain scenarios where a decision is made to employ FDT over continuous therapy, the aim besides improving PFS and OS outcomes, should be to maximise TFI which can in turn, help improve QoL.

Our study is limited by its retrospective nature, lack of QoL data which can further inform of the benefits of TFI, in addition to the use of thalidomide or bortezomib-based therapies which are not reflective of the current shift towards newer therapies and a continuous strategy.

## Conclusion

In conclusion, this large real-world data study details the outcomes of treating TNE NDMM with a FDT approach. First line therapy resulted in the longest TFI which was modest at a median of 6.9 months, and decreased significantly following subsequent lines of therapy. This was demonstrable for the total cohort as well as for individual subgroups by age, and according to co-morbidities. The treatment objective for TNE NDMM should be to maximise the benefit of first line treatment. Continuous therapy has demonstrated its significantly improved survival outcomes, compared to FDT. Therefore, we envisage that the recent shift towards a continuous therapeutic approach will benefit TNE patients, but only if patients are closely monitored with a tolerable regimen. However, when continuous therapy is not appropriate due to patient choice, or toxicities leading discontinuation, an efficacious (not limited to thalidomide or bortezomib) but tolerable FDT strategy remains a reasonable alternative approach, which can produce a meaningful TFI.
